# Convalescent plasma for pediatric patients with SARS-CoV-2-associated acute respiratory distress syndrome

**DOI:** 10.1002/pbc.28693

**Published:** 2020-09-04

**Authors:** Caroline Diorio, Elizabeth M. Anderson, Kevin O. McNerney, Eileen C. Goodwin, Julie C. Chase, Marcus J. Bolton, Claudia P. Arevalo, Madison E. Weirick, Sigrid Gouma, Laura A. Vella, Sarah E. Henrickson, Kathleen Chiotos, Julie C. Fitzgerald, Todd J. Kilbaugh, Audrey R. Odom John, Allison M. Blatz, Michele P. Lambert, Kathleen E. Sullivan, Margaret R. Tartaglione, Danielle Zambrano, Meghan Martin, Jessica H. Lee, Pampee Young, David Friedman, Deborah A. Sesok-Pizzini, Scott E. Hensley, Edward M. Behrens, Hamid Bassiri, David T. Teachey

**Affiliations:** 1 Immune Dysregulation Frontier Program, Department of Pediatrics, Children’s Hospital of Philadelphia, University of Pennsylvania Perelman School of Medicine, Philadelphia, Pennsylvania; 2 Division of Oncology, Department of Pediatrics, Children’s Hospital of Philadelphia, University of Pennsylvania Perelman School of Medicine, Philadelphia, Pennsylvania; 3 Department of Microbiology, Perelman School of Medicine, University of Pennsylvania, Philadelphia, Pennsylvania; 4 Division of Rheumatology, Department of Pediatrics, Children’s Hospital of Philadelphia, University of Pennsylvania Perelman School of Medicine, Philadelphia, Pennsylvania; 5 Division of Infectious Diseases, Department of Pediatrics, Children’s Hospital of Philadelphia, University of Pennsylvania Perelman School of Medicine, Philadelphia, Pennsylvania; 6 Division of Allergy and Immunology, Department of Pediatrics, Children’s Hospital of Philadelphia, University of Pennsylvania Perelman School of Medicine, Philadelphia, Pennsylvania; 7 Division of Critical Care Medicine, Department of Anesthesiology and Critical Care Medicine, Children’s Hospital of Philadelphia, University of Pennsylvania Perelman School of Medicine, Philadelphia, Pennsylvania; 8 Division of Hematology, Children’s Hospital of Philadelphia, University of Pennsylvania Perelman School of Medicine, Philadelphia, Pennsylvania; 9 American Red Cross, Washington, District of Columbia; 10 Pathology and Laboratory Medicine, University of Pennsylvania Perelman School of Medicine, Philadelphia, Pennsylvania

**Keywords:** convalescent plasma, COVID-19, pediatrics, SARS-CoV-2

## Abstract

There are no proven safe and effective therapies for children who develop life-threatening complications of severe acute respiratory syndrome coronavirus 2 (SARS-CoV-2). Convalescent plasma (CP) has demonstrated potential benefit in adults with SARS-CoV-2, but has theoretical risks.We present the first report of CP in children with life-threatening coronavirus disease 2019 (COVID-19), providing data on four pediatric patients with acute respiratory distress syndrome. We measured donor antibody levels and recipient antibody response prior to and following CP infusion. Infusion of CP was not associated with antibody-dependent enhancement (ADE) and did not suppress endogenous antibody response. We found CP was safe and possibly efficacious. Randomized pediatric trials are needed.

## 1 ∣ INTRODUCTION

Early reports from the severe acute respiratory syndrome coronavirus 2 (SARS-CoV-2) global pandemic suggested that children were less severely affected by coronavirus disease 2019 (COVID-19) compared to older adults.^1-3^ Nevertheless, some children with COVID-19 are critically ill.^4,5^ Currently, no therapies have been proven effective in the treatment of COVID-19 in children.

Convalescent plasma (CP) derived from patients that have recovered from SARS-CoV-2 can be infused into currently ill patients. The proposed mechanism of action is via neutralizing antibodies binding to virus, rendering it inert.^6^ Specific to SARS-CoV-2, antibodies against the receptor-binding domain (RBD) have been identified as surrogates for neutralization.^7-11^ CP also contains antibodies against the immunogenic nucleocapsid (N) protein, which are nonneutralizing and present in both actively infected and recovered patients.^7^ The protective function of N antibodies remains unclear.^7,12^ Antibodies against the full-length SARS-CoV-2 spike (S) protein include both nonneutralizing antibodies and neutralizing antibodies.^13^

Initial series of CP in adults with COVID-19 demonstrated potential benefits without apparent side effects.^14-17^ In a recent clinical trial of adults with COVID-19, there was a nonstatistically significant trend toward clinical improvement in CP-treated patients versus controls, without improvement in time to discharge or mortality.^18^ This trial was halted early due to poor accrual.^18^ To date, there have been no reports on the use of CP in children.

CP infusion is associated with the side effects of any blood product: allergic reaction, transfusion-associated circulatory overload, and infection with blood-borne pathogens. There is a theoretical risk that infusion of donor antibodies may impede the recipient’s endogenous production of antibodies, and of antibody-dependent enhancement (ADE), where antibodies developed during a previous infection cause a worsened clinical response with subsequent infection. ADE has been described in dengue fever, and in preclinical models of other coronaviruses.^19-21^ ADE has not been reported in adult patients receiving CP for SARS-CoV-2.^6,17,18^

We present the first report of pediatric patients receiving CP for life-threatening COVID-19-associated respiratory disease with correlative measurements of the associated pre- and posttransfusion antibody response.

## 2 ∣ METHODS

We treated four critically ill children actively infected with SARS-CoV-2 with CP at the Children’s Hospital of Philadelphia (CHOP) under emergency Investigational New Drug applications (eINDs) through the Food and Drug Administration (FDA). Patients were considered for CP if they met FDA guidance for life-threatening disease (Appendix S1). Per the American Red Cross Guidelines, donors were eligible to provide CP if they met the following criteria: (a) they were initially proven positive for SARS-CoV-2 by a laboratory test; and either (b1) at least 14 days from symptom resolution with a repeat documented negative test for SARS-CoV-2, or (b2) at least 28 days from symptom resolution without a documented repeat test results at the time of plasma collection.

We prospectively screened and enrolled these patients into a larger SARS-CoV-2 biobanking protocol. The objective of the SARS-CoV-2 biorepository protocol is to collect samples on patients with confirmed SARS-CoV-2 infection or multisystem inflammatory syndrome in children (MIS-C). An initial report from this biorepository has been published.^22^ Patients were enrolled on the biobank protocol if they had evidence of SARS-CoV-2 infection on reverse-transcriptase polymerase chain reaction (RT-PCR) testing from respiratory tract mucosa. This protocol was approved by the CHOP Institutional Review Board (IRB). Consent was obtained for both protocols from a legal authorized representative per the Declaration of Helsinki (written informed consent for CP; verbal consent for biobanking). Verbal consent for biobanking was obtained based on IRB recommendations to prevent exposure to research staff and to avoid unintentional transmission of SARS-CoV-2 via fomites such as consent forms.

Clinical data were abstracted from patient charts to standardized forms by a research assistant using a REDCap database, and verified by a physician. Blood samples were obtained from participants prior to CP infusion, following CP infusion, and weekly thereafter until death or discharge. Remnant donor CP was retained, per institutional protocol in case of transfusion reaction. Remnant donor CP was then collected and stored.

SARS-CoV-2-specific antibodies were measured by enzyme-linked immunosorbent assay (ELISA) as previously described.^8^ Antibodies in serially diluted plasma (starting at 1:50) were measured against RBD of the SARS-CoV-2 S protein,^9^ full-length S protein, and the N protein (Sino Biological, Wayne, PA). Reciprocal plasma dilutions were calculated from an optical density threshold from a set concentration. Standard curves were generated by diluting an RBD-specific monoclonal antibody (CR3022) starting at 0.5 *μ*g/mL (RBD and S ELISAs), or serially diluted pooled serum from actively SARS-CoV-2-infected adults (N ELISAs). See Appendix S2 for detailed methods.

## 3 ∣ RESULTS AND DISCUSSION

**FIGURE 1:**
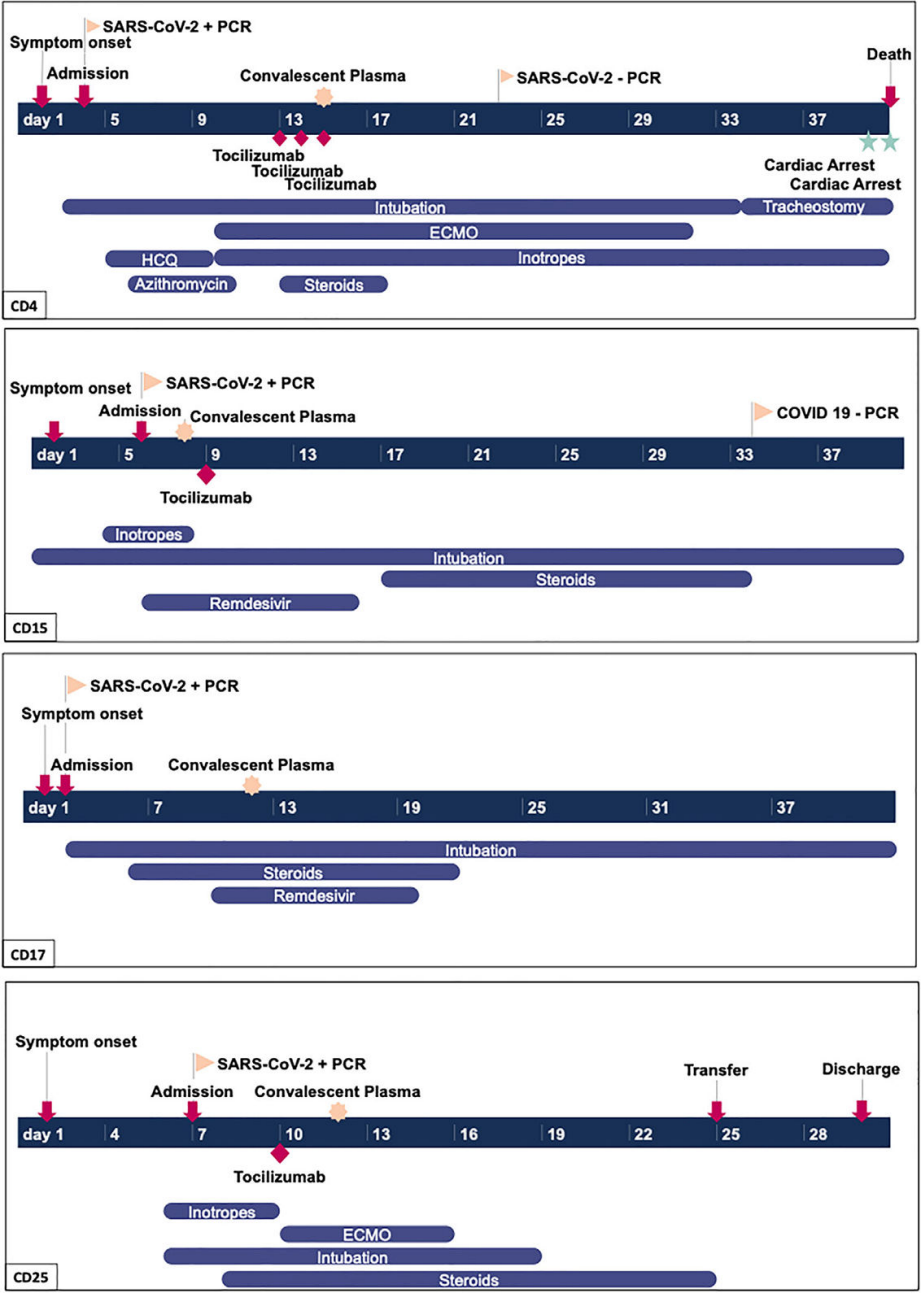
Graphical representation of clinical course of four patients included in the case series. Timeline includes up to day 40 of illness, or until death or discharge, whatever occurred first. ECMO, extracorporeal membrane oxygenation; HCQ, hydroxychloroquine; PCR, polymerase chain reaction

Four patients, 14-18 years, were treated with CP during the study period. Patients received all available plasma from the donors, in the range of 200-220 mL. Patient CD4 received 2 mL/kg, and patients CD15, CD17, and CD25 received 4 mL/kg. All patients were critically ill with COVID-19-associated acute respiratory distress syndrome. All patients required intubation and ventilation; two required extracorporeal membrane oxygenation (ECMO). None of the patients had MIS-C. All patients received other SARS-CoV-2-directed therapies in addition to CP. Graphical case summaries are presented in Figure 1. Further details are presented in Appendix S3. No patients experienced any treatment-emergent adverse events (TEAE) related to CP infusion.

**FIGURE 2:**
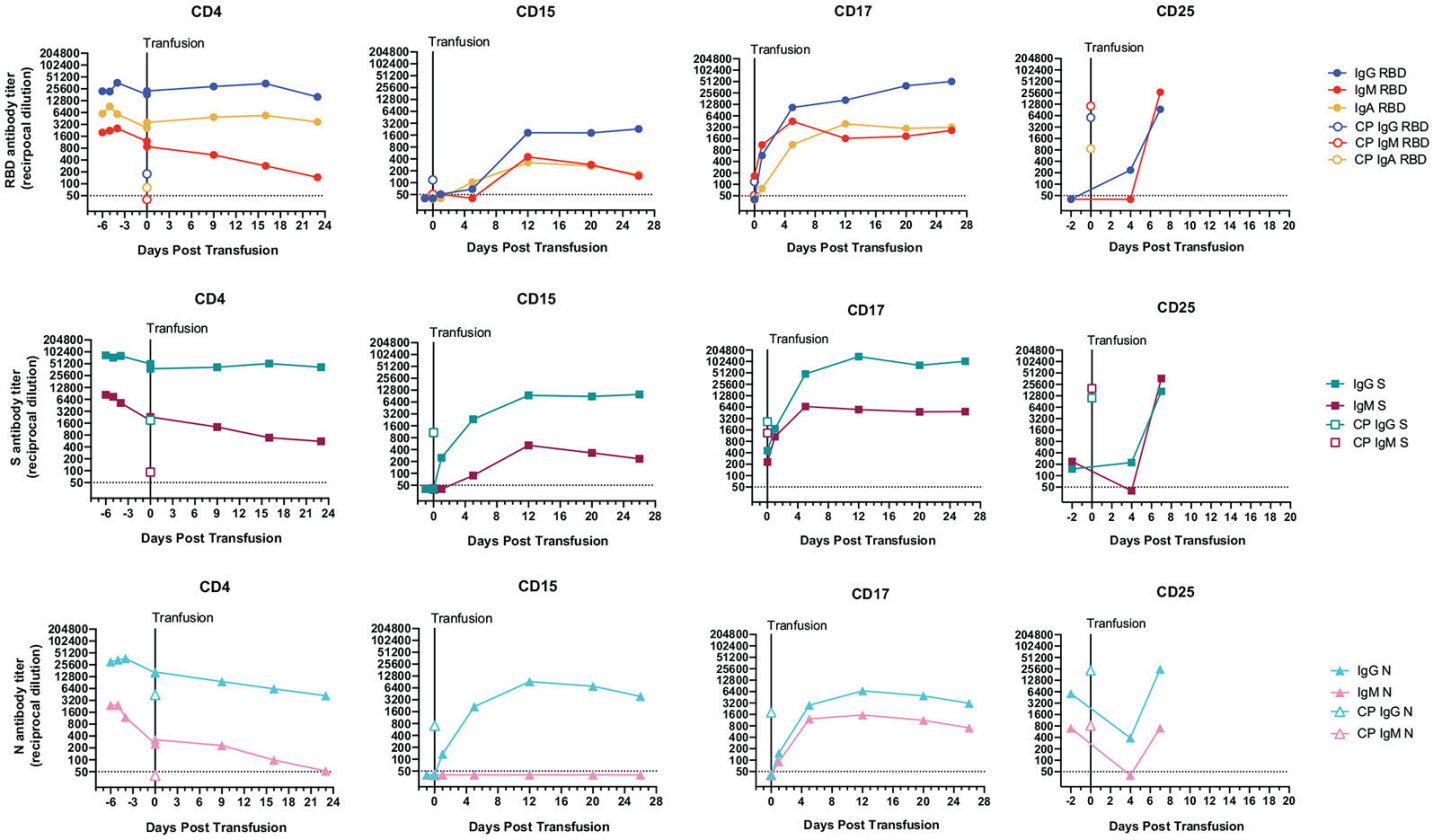
Longitudinal serum SARS-CoV-2 antibody titers in pediatric patients receiving convalescent plasma (CP) transfusion therapy. Antibody titers expressed as reciprocal serum dilution against SARS-CoV-2 antigens in four pediatric patients (CD4, CD15, CD17, and CD25), and corresponding CP donors (open symbols) before and after plasma transfusion. Antibody titers against (A-D) the SARS-CoV-2 receptor-binding domain of the spike (S) protein, (E-H) the full-length SARS-CoV-2 S protein (FL), and (I-L) the nucleoprotein of SARS-CoV-2 (N). Dashed lines denote the lower limit of detection (LLOD) of the assay (1:50 serum dilution)

Longitudinal antibody titers are presented in Figure 2. Corresponding antibody titers for CP donors are displayed. At the time of CP infusion, patient CD4 had extremely high endogenous antibody titers (against SARS-CoV-2 RBD, S, and N). Antibody titers in this participant were sustained posttransfusion. This individual showed transient clinical improvement, decannulating from ECMO; however, died from cardiac causes by day 25 posttransfusion. An autopsy was performed, which demonstrated diffuse alveolar damage and hypertrophic cardiomyopathy with associated myocardial fibrosis. Spleen and lung cultures were positive for *Candida glabrata*. There was no evidence of myocarditis.

Patients CD15, CD17, and CD25 had low or undetectable RBD-specific antibody titers at the time of infusion. Serum antibodies against the SARS-CoV-2 RBD correlate with neutralizing activity, likely due to inhibition of the virus’ ability to bind and enter target cells.^8-10^ Of note, the donor plasma infused into patients CD15 and CD17 had relatively low RBD-specific antibody titer levels (<1:160). Both CP donors for CD15 and CD17 had substantial full-length IgG S titers (>1:1000), and these may have conferred some neutralizing activity. It is unclear if CP with low RBD-specific and high full-length IgG S antibody titers would provide benefit. CD15 and CD17 both remain in intensive care and did not have significant clinical improvement following CP infusion, although CD15 did wean off inotropic support shortly after receiving CP. CD17 did not require inotropic support. Both patients continued to require intubation and ventilatory support despite CP. As of August 22, 2020 both CD15 and CD17 remain in hospital and have had the placement of tracheostomies. They both remain on ventilatory support, although at a lower level than required during acute illness. The donor for patient CD25 had higher SARS-CoV-2 RBD antibody titers (>1:6000) than the donor for any of the other patients, making it more likely that this donor plasma could be beneficial. CD25 had the shortest period of admission and has been discharged from the hospital after being critically ill and on ECMO.

Prior to transfusion, three of the four recipients (CD15, CD17, and CD25) had undetectable IgG RBD antibody levels (<1:50 serum dilution). Upon transfusion, all three participants eventually experienced a boost in IgG, IgM, and IgA antibodies against RBD that was sustained for 7-26 days posttransfusion. In one participant (CD15), we detected antibodies against the full-length S protein before we detected antibodies against the RBD of the S protein. The boost in antibodies against N posttransfusion was similar to that of RBD and S-specific serum antibodies for patients CD15, CD17, and CD25. In all three patients, the serum levels of SARS-CoV-2-specific antibodies eventually surpassed the amount transfused, indicating that these patients mounted a posttransfusion de novo response and that CP did not eliminate the endogenous serological response.

In summary, CP was safely administered to four pediatric patients with severe COVID-19. There were no CP-related TEAE. The theoretical risks of ADE or elimination of endogenous antibody response did not occur. CP may be of greatest benefit for patients who are early in their illness and have not yet generated endogenous antibodies, and when the infused plasma has a high antibody titer. The patient who received CP with the highest antibody titer had the best clinical response, although the total antibody response mounted did not differ markedly from the other patients. The small sample size of our study precludes definitive conclusions about efficacy, however, the excellent clinical response in this patient is encouraging. The other patients had significant comorbidities that may have contributed to their clinical status. It is difficult to determine if CD15 and CD17 benefited, as it is possible without CP they could have continued to worsen. Future research should include randomized controlled trials to definitively examine the efficacy of CP in children.

## Supplementary Material

Online supplement

## Abbreviations:

ADE: antibody-dependent enhancement
CHOP: Children’s Hospital of Philadelphia
COVID-19: coronavirus disease 2019
CP: convalescent plasma
ELISA: enzyme-linked immunosorbent assay
FDA: Food and Drug Administration
MIS-C: multisystem inflammatory syndrome in children
N protein: nucleocapsid protein
RBD: receptor-binding domain
S protein: spike protein
SARS-CoV-2: severe acute respiratory syndrome coronavirus 2
