# Supplementary material for: Convalescent plasma for pediatric patients with SARS-CoV-2-associated acute respiratory distress syndrome
Source: Pediatr Blood Cancer. Author manuscript; Available in PMC 2021 Nov 1. (PMC7734626; doi:10.1002/pbc.28693)
Supplement: Online supplement [file NIHMS1652161-supplement-Online_supplement.pdf]

Supporting Information

Supplemental Appendices, Tables and Figures

Appendix S1. Inclusion Criteria for Convalescent Plasma Based on FDA Guidance
Appendix S2. Details of ELISA Methods.
Appendix S3. Clinical summary of patients included in series.

Appendix S1. Inclusion Criteria for Convalescent Plasma Based on FDA Guidance

Note: The FDA gave guidance on using in severe or life-threatening cases. Given that we included pediatric patients we only used convalescent plasma in children who had life-threatening complications of COVID-19.

1. Laboratory confirmed COVID-19
2. Severe or life-threatening COVID-19, for example
    a. Severe disease is defined as one or more of the following
        i. Shortness of breath (dyspnea),
        ii. Respiratory frequency >= 30/min,
        iii. Blood oxygen saturation <=93%,
        iv. Partial pressure of arterial oxygen to fraction of inspire oxygen ratio <300,
        v. Lung infiltrates >50% within 24 to 48 hours
    b. Life-threatening disease is defined as one or more of the following
        i. Respiratory failure,
        ii. Septic shock,
        iii. Multiple organ dysfunction or failure
3. Informed consent by the patient or healthcare proxy (legally authorized representative)

Appendix S2. Details of ELISA Methods.

ELISA plates were coated overnight at 4°C with 50 μL of SARS-CoV-2 antigen (2μg/well) or PBS (plastic control). Antibodies in serially diluted serum (starting at 1:50) were measured against the receptor binding domain (RBD) of the SARS-CoV-2 spike (S) protein (plasmids were a gift from Florian Krammer, Mt. Sinai)[1], the full-length S protein, and the nucleoprotein (N) (Sino Biological). Coated ELISA plates were washed 3 times with PBS + 0.1% Tween-20 (PBS-T) and blocked for one hour at room temperature with PBS-T supplemented with 3% milk. After blocking, plates were washed 3 times with PBS-T and were incubated for 2 hours at room temperature with 50 μL of diluted serum. HRP-conjugated secondary antibodies were diluted 1:500 (IgA; SouthernBiotech), 1:1000 (IgM; SouthernBiotech), or 1:5000 (IgG; Jackson ImmunoResearch) in PBS-T supplemented with 1% milk (dilution buffer). Plates were again washed 3 times with PBS-T and 50 μL of secondary antibody was incubated for one hour at room temperature. Plates were washed for a final time (3x with PBS-T) before they were developed with the addition of 50 μL of TMB substrate (KPL). The reaction was stopped via acidification after 5 minutes and the optical density (OD) at 450 nm was read on a microplate reader (SpectraMax 190, Molecular devices). Plastic was run in parallel and subtracted from the OD of the sera against antigen. Reciprocal serum dilutions were calculated from an OD threshold from a set concentration on a standard curve that was included on each plate. Standard curves were generated by diluting an RBD-reactive monoclonal antibody (CR3022; a gift from Ian Wilson, Scripps Research Institute) starting at 0.5 μg/mL (RBD and S ELISAs) or serially diluted pooled serum from actively SARS-CoV-2 infected adults (N ELISAs).

Appendix S3. Clinical summary of patients included in series.

ID	Age, Sex	Comorbidities	Day of CP infusion*	ARDS?	Inotropes required?	ECMO?	Thrombotic complications	Co-infections	Other COVID-19 Therapies Received	Outcome
**CD4**	18, F	Hypertension, IDDM, obesity	14	Yes	Yes	Yes	None diagnosed, enoxaparin prophylaxis	*C. glabrata* respiratory culture positive on day 27 of illness	Tocilizumab, hydroxychloroquine, corticosteroids	Died on day 40 of illness
**CD15**	15, F	Epilepsy, developmental regression, home BiPAP requirement	7	Yes	Yes	No	None diagnosed, enoxaparin prophylaxis	*E. coli* UTI on day 43 of illness; P. aeruginosa respiratory colonization	Remdesivir, tocilizumab	Remains in hospital with tracheostomy
**CD17**	17, F	Spastic quadriplegia, encephalopathy; intermittent home BiPAP	11	Yes	No	No	Line associated thrombus, treated with enoxaparin	*K. pneumoniae* UTI on day 40 of illness	Remdesivir, corticosteroids	Remains in hospital with tracheostomy
**CD25**	14, F	<28 weeks gestation; no long-term sequelae of prematurity	11	Yes	Yes	Yes	None diagnosed, enoxaparin prophylaxis	None	Tocilizumab, corticosteroids	Recovered

* from symptom onset; BiPAP – bilevel positive airway pressure; *C. glabrata – Candida glabrata*; E. Coli – *Eschericii coli*; F – female; IDDM – insulin dependent diabetes mellitus; K. pneumoniae – klebsiella pneumoniae; UTI – urinary tract infection; CD4 – eIND 20164; CD15 – eIND 2180; CD17 – eIND 21798; CD25 – eIND 22389

References

1. Amanat F, Stadlbauer D, Strohmeier S, et al. A serological assay to detect SARS-CoV-2 seroconversion in humans. *Nat Med*. 2020.
